# Comparative Efficacy and Safety of Cardio-Renoprotective Pharmacological Interventions in Chronic Kidney Disease: An Umbrella Review of Network Meta-Analyses and a Multicriteria Decision Analysis

**DOI:** 10.3390/biom15010039

**Published:** 2024-12-31

**Authors:** Ioannis Bellos, Smaragdi Marinaki, Pagona Lagiou, Vassiliki Benetou

**Affiliations:** 1 Department of Hygiene, Epidemiology and Medical Statistics, Medical School, National and Kapodistrian University of Athens, 75, Mikras Asias Str., 115 27 Athens, Greecevbenetou@med.uoa.gr (V.B.); 2 Department of Nephrology and Renal Transplantation, Laiko General Hospital, Medical School, National and Kapodistrian University of Athens, 75, Mikras Asias Str., 115 27 Athens, Greece; smaragdimarinaki@yahoo.com

**Keywords:** chronic kidney disease, sglt2, glp1, finerenone, network meta-analysis

## Abstract

Sodium-glucose co-transporter 2 inhibitors (SGLT2i), glucagon-like peptide-1 receptor agonists (GLP1a), and non-steroidal mineralocorticoid receptor antagonists (ns-MRA) are promising treatments for chronic kidney disease. This umbrella review of network meta-analyses evaluated their effects on cardiovascular outcomes, kidney disease progression, and adverse events, using the TOPSIS method to identify the optimal intervention based on P-scores. A total of 19 network meta-analyses and 44 randomized controlled trials involving 86,150 chronic kidney disease patients were included. Compared to placebo, SGLT2i were associated with reduced risks of cardiovascular events [Hazard ratio (HR): 0.776, 95% confidence intervals (CI): 0.727–0.998], kidney disease progression (HR: 0.679, 95% CI: 0.629–0.733), acute kidney injury (HR: 0.873, 95% CI: 0.773–0.907), and serious adverse events (HR: 0.881, 95% CI: 0.847–0.916). GLP1a and ns-MRA were also associated with significant reductions in cardiovascular and kidney-specific composite outcomes. Indirect evidence showed that SGLT2i demonstrated a lower risk of kidney disease progression compared to GLP1a (HR: 0.826, 95% CI: 0.716–0.952) and ns-MRA (HR: 0.818, 95% CI: 0.673–0.995), representing the best intervention across all endpoints. In conclusion, while SGLT2i, GLP1a, and ns-MRA all reduce cardiovascular and kidney disease risks in chronic kidney disease, SGLT2i appears to provide the most favorable balance of efficacy and safety.

## 1. Introduction

Chronic kidney disease represents a rising global concern, affecting more than 10% of the population worldwide [1]. It is one of the main non-communicable causes of premature mortality and is associated with reduced quality of life, disability, and high psychological and economic costs [2]. Cardiovascular disease constitutes the leading cause of death among chronic kidney disease patients, due to the interplay of traditional risk factors, especially diabetes mellitus and hypertension, with non-traditional factors specific to kidney dysfunction, such as inflammation, mineral bone disease, and proteinuria [3]. In this context, it has been estimated that in 2017, chronic kidney disease led to more than 1.4 million cardiovascular deaths and the loss of 25 million cardiovascular disability-adjusted life years (DALYs) [4]. As a result, effective interventions are needed to reduce the burden of chronic kidney disease, decrease the risk of cardiovascular disease, and prevent the progression of kidney failure, necessitating renal replacement therapy.

In recent years, research has focused on developing or repurposing novel therapeutic approaches, aiming to limit progressive loss of renal function and reduce the risk of chronic kidney disease complications [5]. Novel antidiabetics, especially sodium-glucose co-transporter 2 inhibitors (SGLT2i) and glucagon-like peptide-1 receptor agonists (GLP1a) have gained increasing interest due to their potential cardioprotective and renoprotective effects among patients with and without diabetes mellitus. From a pathophysiological point of view, SGLT2i are able to restore the tubuloglomerular feedback mechanism alleviating glomerular hyperfiltration, as well as to improve renal tissue energy supply, limit oxidative stress and inhibit pro-inflammatory and pro-fibrotic pathways [6]. On the other hand, apart from enhancing glycemic control and promoting bodyweight loss, GLP1a have been proposed to increase natriuresis via their inhibitory action on the sodium-hydrogen exchanger 3 of the proximal tubule, limit the generation of reactive oxygen species and the production of pro-inflammatory cytokines [7]. In addition, finerenone, a highly selective and potent non-steroidal mineralocorticoid receptor antagonist (ns-MRA) has been suggested to reverse endothelial dysfunction and albuminuria, reduce oxidative stress, inhibit cardiac fibrosis, and improve left ventricular function [8,9].

A growing number of network meta-analyses have been recently published, aiming to summarize the effects of SGLT2i, GLP1a, and ns-MRA in chronic kidney disease and provide indirect evidence about their relative efficacy and safety. However, heterogeneity in design, eligibility criteria, and statistical methodology may generate conflicting results and complicate the drawing of clinically useful conclusions [10]. The present umbrella review evaluated the existing network meta-analyses in the field and provided pooled estimates comparing the effects of SGLT2i, GLP1a, and ns-MRA in chronic kidney disease, with and without diabetes mellitus. In addition, a multicriteria decision analysis has been implemented as a novel approach to generate rankings of the above interventions evaluating multiple potentially conflicting endpoints in parallel, aiming to provide guidance about the choice of the optimal treatment based on clinical priorities.

## 2. Materials and Methods

### 2.1. Study Design

The study protocol has been prospectively registered and is publicly available (https://dx.doi.org/10.17504/protocols.io.81wgbrdx3lpk/v1 accessed on 29 December 2024). The network meta-analysis has been reported following the PRISMA-NMA (Preferred Reporting Items for Systematic reviews and Meta-Analyses extension for systematic reviews incorporating network meta-analysis) guidelines [11]. No ethical approval was required since the study was exclusively based on already published data.

### 2.2. Eligibility Criteria

The study population consisted of patients with chronic kidney disease as defined by the KDIGO (Kidney Disease: Improving Global Outcomes) guidelines [12]. In particular, the diagnosis of chronic kidney disease was based on the presence of an estimated glomerular filtration rate (eGFR) below 60 mL/min/1.73 m^2^ and/or a urinary albumin-to-creatinine ratio (UACR) greater than 30 mg/g. Studies on patients with and without diabetes mellitus could be eligible for inclusion. The evaluated interventions included SGLT2i, GLP1a, and nsMRA, while the placebo served as a comparator. The efficacy outcomes of interest consisted of the occurrence of major cardiovascular events and kidney disease progression, as defined by the cardiovascular and kidney-specific composite endpoints of each randomized controlled trial (RCT). The safety endpoints included the risk of acute kidney injury, any serious adverse event and any adverse event leading to permanent drug discontinuation. The umbrella review was based on network meta-analyses of RCTs, including at least one of the interventions under investigation. Pairwise meta-analyses or meta-analyses of observational studies were not evaluated. Nonrandomized interventions (e.g., interventions administered at baseline prior to randomization) were also not taken into account.

### 2.3. Literature Search

The following databases were systematically searched from inception to 1 October 2024: Medline, Scopus, Web of Science, and Cochrane Central Register of Controlled Trials (CENTRAL). An additional search through Google Scholar was performed, aiming to identify possible missing network meta-analyses. The main search algorithm was the following: “((“Glucagon-Like Peptide-1 Receptor Agonists” [Mesh] OR “Glucagon-Like Peptide 1” [Mesh] OR GLP-1 OR “Glucagon-Like Peptide-1 agonist*” OR exenatide OR exendin OR liraglutide OR albiglutide OR dulaglutide OR lixisenatide OR semaglutide OR taspoglutide OR tirzepatide) OR (“Sodium-Glucose Transporter 2 Inhibitors” [Mesh] OR “sodium-glucose cotransporter-2 inhibitor*” OR “sglt2 inhibitor*” OR “sglt-2 inhibitor*” OR gliflozin OR canagliflozin OR bexagliflozin OR dapagliflozin OR empagliflozin OR ertugliflozin OR ipragliflozin OR licogliflozin OR luseogliflozin OR remogliflozin OR sergliflozin OR sotagliflozin OR tofogliflozin OR henagliflozin) OR (“Mineralocorticoid Receptor Antagonists” [Mesh] OR spironolactone OR canrenone OR finerenone OR mexrenone) OR (“Renal Insufficiency, Chronic” [Mesh] OR chronic kidney disease OR nephropathy OR chronic renal insufficiency OR chronic renal impairment OR CKD)) AND (“Network Meta-Analysis” [Mesh] OR “network meta-analysis” OR “network metaanalysis”)”. No date or language restrictions were applied.

### 2.4. Study Selection

The selection of network meta-analyses was performed through a 3-step process. At first, the titles and abstracts of all electronic records were screened for eligibility. Subsequently, all articles presumed to meet the inclusion criteria were retrieved as full texts. Then, after reviewing the full texts, the final included network meta-analyses were selected based on the predefined inclusion and exclusion criteria. The original RCTs were identified by manually searching the reference lists of the included network meta-analyses. In case of missing data regarding study characteristics or outcomes of interest, additional reports of the examined RCT were identified through Medline search. The study selection process was independently conducted by two reviewers, resolving any discrepancies through the consensus of all authors.

### 2.5. Data Collection

The following parameters were extracted from the included network meta-analyses: year of publication, country, type of population (presence of diabetes mellitus), network nodes, statistical model (frequentist or Bayesian), evaluation of transitivity, assessment of heterogeneity, treatment ranking, certainty of evidence evaluation, funding, and presence of a pre-registered protocol. The network density expressed the connectedness of the network graph and was estimated as the total number of connections divided by the number of possible connections, ranging from 0 to 1 [13]. The information regarding the methodology of the original RCTs included the following: trial name or registration number, year of publication, type of population (presence of chronic kidney disease), examined intervention, sample size, mean age, percentage of females, mean body mass index, percentage of diabetes mellitus and cardiovascular disease history, mean eGFR, and UACR. Regarding the outcomes of interest, hazard ratios (HR) along with their 95% confidence intervals (CI) or the number of events in the total number of patients within each study arm were extracted. Different reports of the same RCT were included to extract estimates for distinct endpoints or specific patient subgroups. Consequently, no duplicate estimates were utilized in the analyses. Two authors independently collected data and any potential differences were resolved through the consensus of all authors.

### 2.6. Quality Assessment

The risk of bias in the included network meta-analyses was evaluated by combining the ROBIS [14] with the ROB-NMA tool [15]. In particular, the ROBIS tool was used to assess the risk of bias in systematic reviews by covering the domains of study eligibility criteria, identification and selection of studies, data collection, and study appraisal. The ROB-NMA tool was applied as a more specific tool for the quality assessment of network meta-analyses, as it takes into account the domains of interventions and network geometry, effect modification, and synthesis. The overall risk of bias could be judged as low, moderate, or high. On the other hand, the risk of bias in the original RCTs was assessed using the RoB-2 tool [16], evaluating the following domains: randomization process, deviations from intended interventions, missing outcome data, measurement of the outcome, and selection of the reported result. The risk of bias evaluation was performed by two reviewers, resolving any disagreements through the consensus of all authors.

### 2.7. Data Analysis

Network meta-analysis was performed by pooling the data derived from the original RCTs. A frequentist methodology was followed and random-effects statistical models were fitted by assuming a common heterogeneity parameter across comparisons. The plausibility of the transitivity assumption was evaluated by examining the distribution of important covariates (age, female sex, body mass index, eGFR, and UACR) across interventions. The effect measure was HR for efficacy outcomes and risk ratio (RR) for safety outcomes. Specifically, the HR or RR extracted from each included study were log-transformed, and the corresponding standard errors were calculated based on the reported 95% CI. For safety endpoints, when the RR was not directly reported, it was derived from the data provided in the 2 × 2 contingency table. Statistical significance was defined at the level of 5%. The construction of league tables enabled the visualization of the relative effects of interventions. The ranking of interventions was performed through their estimated P-scores for each endpoint, with higher P-score values indicating better treatments. Subgroup analyses were also conducted by separately examining studies at low risk of bias, patients with diabetes mellitus, patients with cardiovascular disease, those with an eGFR < 45 mL/min/1.73 m^2^ or an eGFR < 30 mL/min/1.73 m^2^, as well as patients with UACR > 300 mg/g.

A multicriteria decision analysis was performed to indicate the optimal compromise intervention, taking into account the calculated P-scores for the efficacy and safety endpoints [17]. Specifically, the distance-based TOPSIS (Technique for Order of Preference by Similarity to Ideal Solution) method [18] was implemented to select the best treatment as the one with the maximum distance from the positive ideal point and the minimum distance from the negative ideal one. Weights were assigned to endpoints both objectively and subjectively. In particular, the entropy method was used as an objective approach that assigns higher weights to outcomes with higher P-score dispersion [18]. On the other hand, the analytical hierarch process was implemented as a subjective method for weight determination [19]. To this end, pairwise comparison matrices were constructed by converting verbal evaluations to numerical integer values (1 to 9), with higher values being indicative of the stronger importance of one endpoint over another. Three different scenarios were examined, aiming to reflect different clinical priorities by assigning more importance to the cardiovascular composite endpoint, the renal endpoints (kidney-specific composite endpoint and acute kidney injury risk), and the safety outcomes (risk of serious adverse events and drug discontinuation). In the first scenario, the cardiovascular composite endpoint was considered three times more important than the kidney-specific composite endpoint and 5–9 times more important than the safety outcomes. In the second scenario, the kidney-specific composite outcome and the incidence of acute kidney injury were regarded as five times more significant than the cardiovascular composite endpoint. In the third scenario, the greatest importance was attributed to the drug discontinuation endpoint, which was judged to be nine times more critical than both the cardiovascular and kidney-specific composite outcomes. Consistency ratios below 10% indicated acceptable consistency of the constructed matrices [20]. All analyses were conducted using R version 4.4.1. (main packages: “metafor” [21] and “topsis” [22]).

### 2.8. Certainty of Evidence

The certainty of the existing evidence was evaluated following the Confidence in Network Meta-Analysis (CiNeMA) method [23], which follows the Grading of Recommendations Assessment, Development and Evaluation (GRADE) approach [24] and takes into account the following domains: within-study bias, reporting bias, indirectness, imprecision, heterogeneity and incoherence. Two reviewers independently evaluated the certainty of evidence using the CiNeMA tool and any discrepancies were resolved through the consensus of all authors.

## 3. Results

### 3.1. Study Selection

The process of study selection is schematically depicted in Supplementary Figure S1 (Section S1). After deduplication, 1154 electronic records were screened and 20 of them were retrieved as full texts. Consequently, one study [25] was excluded for implementing a different methodology, and as a result, 19 network meta-analyses [26,27,28,29,30,31,32,33,34,35,36,37,38,39,40,41,42,43,44] were included in the present review. At the next step, original studies were identified, including 64 reports [45,46,47,48,49,50,51,52,53,54,55,56,57,58,59,60,61,62,63,64,65,66,67,68,69,70,71,72,73,74,75,76,77,78,79,80,81,82,83,84,85,86,87,88,89,90,91,92,93,94,95,96,97,98,99,100,101,102,103,104,105,106,107,108] of 44 RCTs, with a total sample size of 86,150 chronic kidney disease patients.

### 3.2. Included Network Meta-Analyses

Table 1 presents the methodological characteristics of the included network meta-analyses. The country of origin was China in 12 studies, Japan in 4 studies, USA in 2 studies, and Canada in 1 study. Non-diabetic patients with chronic kidney disease were included in three meta-analyses. The median number of nodes was 4 (range: 3 to 14) and the median density plot was 0.5 (range: 0.18 to 1). The nodes consisted of drug categories and individual drugs in 13 and 6 network meta-analyses, respectively. The median number of included RCTs per network meta-analysis was 14 (range: 3 to 29). Regarding the statistical methodology, 12 network meta-analyses used frequentist models, 6 used Bayesian models, and 1 implemented both approaches. The plausibility of the transitivity assumption was tested in 3 studies, while heterogeneity was evaluated in 12 studies through subgroup and/or sensitivity analyses. A ranking of interventions was provided in 12 network meta-analyses reporting P-scores or SUCRA (surface under the cumulative ranking curve) values, while the certainty of evidence was appraised in four studies. Sixteen meta-analyses mentioned pre-registered protocols and ten received any form of funding (industry in three and non-profit in seven studies). The outcomes of the quality assessment are displayed in Table 2, indicating a high risk of bias in 18 and a moderate risk of bias in 2 studies. The ROBIS tool identified mainly concerns of bias in the domain of study eligibility criteria (12 studies) and that of data collection and study appraisal (14 studies) due to inadequacies in patient inclusion criteria description, outcome definitions, and risk of bias evaluation. In addition, the ROB-NMA tool recognized concerns of bias mainly in the domains of effect modification (all studies) and synthesis (17 studies), coming from the lack of appropriate assessment of transitivity and heterogeneity via subgroup or meta-regression analyses.

### 3.3. Included RCTs

The methodological characteristics of the included RCTs are summarized in Supplementary Table S1 (Section S2). SGLT2i were evaluated in 28, GLP1a in 11 and ns-MRA in 5 RCTs. In all studies, the above interventions were compared to the placebo, resulting in a star-shaped network (Supplementary Figure S2). Chronic kidney disease patients were exclusively included in 23 trials, while 21 RCTs reported subgroup analyses of participants with chronic kidney disease. Non-diabetic patients were included in five RCTs, while a history of cardiovascular disease was a prerequisite for inclusion in ten trials. The definitions of kidney-specific and cardiovascular composite endpoints are presented in Supplementary Table S2 (Section S2). According to the RoB-2 tool, a moderate risk of bias was recognized in 27 RCTs, with concerns of bias arising mainly from the domain of randomization, especially in studies reporting subgroup analyses of chronic kidney disease patients (Supplementary Table S3). Section S4 (Supplementary Figures S3–S6) shows the comparisons of baseline patient characteristics across interventions, suggesting that GLP1a-treated patients presented higher body mass index, while those treated with ns-MRA had a higher percentage of females and lower body mass index.

### 3.4. Cardiovascular Composite Endpoint

**Figure 1:**
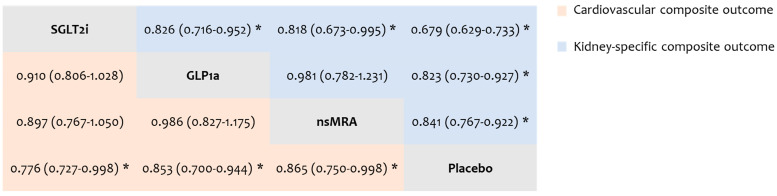
League table comparing interventions for the risk of the cardiovascular composite (lower half) and kidney-specific composite (upper half) outcome. Each cell presents the relative effects of interventions as hazard ratios with 95% confidence intervals. Statistically significant differences are indicated by asterisks.

The cardiovascular composite outcome was assessed in 24 RCTs. Compared to patients receiving placebo, the risk of reaching the cardiovascular composite endpoint was significantly lower in patients treated with SGLT2i (HR: 0.776, 95% CI: 0.727 to 0.998, low certainty), GLP1a (HR: 0.853, 95% CI: 0.700 to 0.944, moderate certainty), and ns-MRA (HR: 0.865, 95% CI: 0.750 to 0.998, moderate certainty). No significant differences were observed in the indirect comparisons among the above interventions (very low to low certainty) (Figure 1). The ranking of treatments indicated SGLT2i as the best (P-score: 0.949), followed by GLP1a (P-score: 0.543), ns-MRA (P-score: 0.500), and placebo (P-score: 0.008). The outcomes of the CiNeMA evaluation are shown in Supplementary Table S4 (Section S5).

### 3.5. Kidney-Specific Composite Endpoint

The kidney-specific composite outcome was evaluated in 19 RCTs. Compared to placebo, the risk of reaching the kidney-specific composite endpoint was significantly lower with SGLT2i (HR: 0.679, 95% CI: 0.629 to 0.733, low certainty), GLP1a (HR: 0.823, 95% CI: 0.730 to 0.927, high certainty), and ns-MRA (HR: 0.841, 95% CI: 0.767 to 0.922, low certainty). In addition, SGTL2i therapy was associated with a significantly lower risk of the kidney-specific composite outcome indirectly compared to GLP1a (HR: 0.826, 95% CI: 0.716 to 0.952, low certainty) and ns-MRA (HR: 0.818, 95% CI: 0.673 to 0.995, low certainty) (Figure 1). The ranking of interventions indicated SGLT2i as the best (P-score: 0.983), followed by GLP1a (P-score: 0.529), ns-MRA (P-score: 0.479), and placebo (P-score: 0.010). The outcomes of the CiNeMA assessment are presented in Supplementary Table S5 (Section S6).

### 3.6. Safety Endpoints

#### 3.6.1. Serious Adverse Events

**Figure 2:**
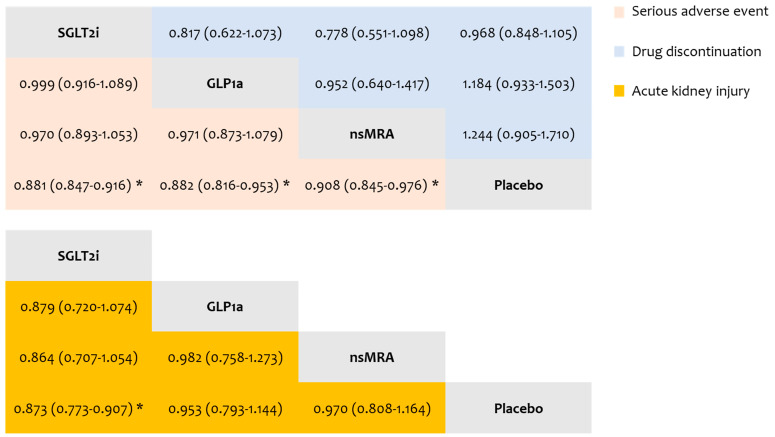
League tables comparing interventions for the risk of serious adverse events, adverse events leading to drug discontinuation, and acute kidney injury. Each cell presents the relative effects of interventions as hazard ratios with 95% confidence intervals. Statistically significant differences are indicated by asterisks.

The outcome of serious adverse events was evaluated in 27 RCTs. Compared to the placebo, the risk of serious adverse events was significantly lower with SGLT2i (HR: 0.881, 95% CI: 0.847 to 0.916, low certainty), GLP1a (HR: 0.882, 95% CI: 0.816 to 0.953, low certainty), and ns-MRA (HR: 0.908, 95% CI: 0.845 to 0.976, low certainty). No significant differences were detected in the indirect comparisons among SGLT2i, GLP1a and ns-MRA (very low to low certainty) (Figure 2). The ranking of interventions suggested that the best treatment was SLGT2i (P-score: 0.760), followed by GLP1a (P-score: 0.732), ns-MRA (P-score: 0.506), and placebo (P-score: 0.002). The results of the CiNeMA evaluation are presented in Supplementary Table S6 (Section S7).

#### 3.6.2. Adverse Events Leading to Drug Discontinuation

The endpoint of drug discontinuations due to adverse events was examined in 32 RCTs. No significant differences were observed both in direct and indirect comparisons (very low to low certainty) (Figure 2). In treatment ranking, SGLT2i emerged as the best intervention (P-score: 0.845), followed by placebo (P-score: 0.715), GLP1a (P-score: 0.250) and ns-MRA (P-score: 0.190). The outcomes of the CiNeMA evaluation are shown in Supplementary Table S7 (Section S7).

#### 3.6.3. Acute Kidney Injury

The endpoint of acute kidney injury was assessed in 29 RCTs. Compared to placebo, SGLT2i therapy was associated with a significantly lower risk of acute kidney injury (HR: 0.873, 95% CI: 0.773 to 0.907, high certainty). No significant differences were noted in the other direct (low certainty) and indirect (very low to low certainty) comparisons (Figure 2). SGTL2i presented the highest ranking (P-score: 0.941), followed by GLP1a (P-score: 0.452), ns-MRA (P-score: 0.384) and placebo (P-score: 0.224). The results of the CiNeMA evaluation are presented in Supplementary Table S8 (Section S7).

### 3.7. Subgroup Analyses

#### 3.7.1. Diabetes Mellitus

In patients with diabetes mellitus, the risk of the cardiovascular composite outcome was significantly lower with SGLT2i (HR: 0.768, 95% CI:0.698 to 0.846) and GLP1a (HR: 0.850, 95% CI: 0.760 to 0.951) treatment, compared to placebo (Supplementary Figure S8, Section S8). SGLT2i emerged as the best treatment (P-score: 0.932). Regarding the kidney-specific composite outcome, all interventions were superior to placebo; however, SGLT2i therapy (P-score: 0.999) was associated with a significantly lower risk of reaching the endpoint compared to treatment with GLP1a (HR: 0.762, 95% CI: 0.655 to 0.886) and ns-MRA (HR: 0.745, 95% CI: 0.654 to 0.850).

#### 3.7.2. Cardiovascular Disease History

Pooling the studies that included patients with cardiovascular disease at baseline suggested that SGLT2i therapy was associated with a significantly lower risk of the cardiovascular composite outcome (HR: 0.793, 95% CI: 0.717 to 0.877) (Supplementary Figure S9) and was ranked as the best intervention (P-score: 0.975). No significant difference was noted between SGLT2i and placebo in regard to the kidney-specific composite outcome (HR: 0.818, 95% CI: 0.624 to 1.072).

#### 3.7.3. Chronic Kidney Disease Stage ≥ 3b

In patients with eGFR < 45 mL/min/1.73 m^2^, SLGT2i administration was associated with a significantly lower risk of the cardiovascular composite outcome, compared to GLP1a therapy (HR: 0.745, 95% CI: 0.560 to 0.989) and placebo (HR: 0.801, 95% CI: 0.732 to 0.878) (Supplementary Figure S10); hence, SGLT2i emerged as the best intervention (P-score: 0.972). Compared to the placebo, a lower risk of the kidney-specific composite outcome was observed for SGLT2i (HR: 0.717, 95% CI: 0.631 to 0.815) and ns-MRA (HR: 0.820, 95% CI:0.712 to 0.945). The ranking of interventions indicated SGLT2i as the best treatment (P-score: 0.914). In patients with eGFR < 30 mL/min/1.73 m^2^, compared to the placebo, a lower risk of the cardiovascular composite outcome was estimated for SGLT2i (HR: 0.776, 95% CI: 0.619 to 0.972) and ns-MRA (HR: 0.430, 95% CI: 0.204 to 0.906) (Supplementary Figure S11). The intervention with the highest ranking was ns-MRA (P-score: 0.905, followed by SGLT2i (P-score: 0.503). In this population, SGLT2i administration was linked to a significantly lower risk of the kidney-specific composite outcome (HR: 0.701, 95% CI: 0.607 to 0.810), while no evidence exists regarding the other interventions.

#### 3.7.4. Macroalbuminuria

In patients with UACR > 300 mg/g, compared to placebo, a lower risk of the cardiovascular composite outcome was observed with SGLT2i (HR; 0.665, 95% CI: 0.589 to 0.750) and ns-MRA (HR: 0.874, 95% CI: 0.778 to 0.982) therapy. SGLT2i was superior to GLP1a (HR: 0.730, 95% CI: 0.550 to 0.970) and ns-MRA (HR: 0.761, 95% CI: 0.643 to 0.899) (Supplementary Figure S12) and as a result, SGLT2 emerged as the best intervention (P-score: 0.995). Regarding the kidney-specific composite outcome, a lower risk of reaching the endpoint was noted for patients treated with SGLT2i (HR: 0.629, 95% CI: 0.562 to 0.918) and ns-MRA (HR: 0.802, 95% CI: 0.701 to 0.905). SGLT2i were estimated to be superior to ns-MRA (HR: 0.784, 95% CI: 0.658 to 0.933) and thus, emerged as the best intervention (P-score: 0.998).

#### 3.7.5. Low Risk of Bias

The results of the network meta-analysis of RCTs at low risk of bias are presented in Supplementary Figure S13. Compared to placebo, a lower risk of the cardiovascular composite outcome was estimated for therapy with SGLT2i (HR: 0.738, 95% CI: 0.667 to 0.816), GLP1a (HR: 0.853, 95% CI: 0.743 to 0.979), and ns-MRA (HR: 0.865, 95% CI: 0.788 to 0.951). SGLT2i administration was superior to ns-MRA therapy (HR: 0.853, 95% CI: 0.743 to 0.979) and emerged as the best intervention (P-score: 0.943). On the other hand, compared to the placebo, a lower risk of the kidney-specific cardiovascular outcome was calculated for SGLT2i (HR: 0.655, 95% CI: 0.596 to 0.721), GLP1a (HR: 0.790, 95% CI: 0.660 to 0.946), and ns-MRA (HR: 0.841, 95% CI: 0.764 to 0.925). Indirect comparisons suggested that SGLT2i therapy was linked to a significantly lower risk of the kidney-specific composite outcome, compared to ns-MRA administration (HR: 0.779, 95% CI: 0.681 to 0.892). The ranking of treatments indicated SGLT2i as the best intervention (P-score: 0.988).

### 3.8. Multicriteria Decision Analysis

The outcomes of the multicriteria decision analysis are presented in Table 3. The analysis assigning equal weights to the examined outcomes (cardiovascular composite outcome, kidney-specific composite outcome, acute kidney injury, any serious adverse event, and drug discontinuation due to adverse event) indicated that treatment with SGLT2i had the highest TOPSIS score (1) and ranked first, followed by GLP1a (TOPSIS score: 0.517), ns-MRA (TOPSIS score: 0.429), and placebo (TOPSIS score: 0.240). Similar outcomes were obtained when weights were assigned using the objective entropy method. The comparison matrices used for subjective weight determination with the analytical hierarchical process are shown in Supplementary Table S9. For the three scenarios, the consistency ratios were 2.8%, 7.0%, and 2.7%, respectively, corresponding to consistent matrices. In all scenarios, SGTL2i emerged as the optimal treatment (TOPSIS score: 1). When more importance was placed on the cardiovascular composite outcome (scenario 1) or the kidney outcomes (scenario 2), GLP1a ranked second, followed by ns-MRA and placebo. In the third scenario placing more importance on safety outcomes, the placebo ranked second (TOPSIS score: 0.484), followed by GLP1a (TOSPIS score: 0.437) and ns-MRA (TOPSIS score: 0.321).

## 4. Discussion

The present umbrella review gathered the existing network meta-analyses, aiming to shed more light on the efficacy and safety of SGLT2i, GLP1a, and ns-MRA in chronic kidney disease. Through the evaluation of 19 network meta-analyses, 44 RCTs were identified, providing a total sample size of 86,150 patients with chronic kidney disease. The pooling of evidence indicated that the three examined interventions were associated with a significantly lower risk of cardiovascular and kidney-specific composite outcomes. All interventions presented a favorable safety profile, as their administration was associated with a lower risk of any serious adverse event, compared to placebo. SGLT2i therapy was suggested to offer the greatest renoprotection, as it was associated with a reduced risk of the kidney-specific composite outcome, compared to both GLP1a and ns-MRA. In addition, despite the theoretical concerns of kidney function deterioration following SGLT2i initiation, their administration was linked to a significantly lower acute kidney risk. This finding has been confirmed by large-scale observational studies [109,110], corroborating the kidney-protective properties of SGTL2i, which may be potentially mediated by the amelioration of renal cortical oxygenation, the attenuation of ischemia–reperfusion injury, the decrease in inflammation and the suppression of fibrosis [111,112,113].

The overview of overlapping network meta-analyses in the field revealed significant differences in the definition of nodes and the selection of RCTs, despite the target population being the same and the research questions being similar. Although 44 RCTs are currently available, most network meta-analyses have included less than one-third of them. The erratic selection of RCTs and the varying definitions of nodes (as drug categories or individual drugs) may increase the degrees of freedom, potentially leading to vibration of effects across different meta-analyses [114]. It should be noted that the majority of the examined network meta-analyses come from China, although most RCTs have been conducted in non-Asian populations. Overall, the risk of bias in the evaluated network meta-analyses was high, reflecting among others, the lack of appropriate assessment of transitivity and heterogeneity. Three network meta-analyses [28,34,41] reported any kind of industry funding. Interestingly, two [28,41] evaluated the combination of SGLT2i and finerenone by including a FIDELITY analysis [115] of patients receiving SGLT2i at baseline (FIDELIO-DKD and FIGARO-DKD trials). Both studies concluded that the combination therapy was superior to monotherapy with finerenone, a finding that contradicts the results of the original study. The incoherent networks of selected RCTs could lead to distorted effect estimates responsible for the observed contradiction.

This study implemented a multicriteria decision analysis as an additional clinically useful tool to guide optimal treatment selection, balancing efficacy and safety. SGLT2i therapy emerged as the intervention of choice in all scenarios since SGLT2i administration ranked first in all efficacy and safety endpoints. Importantly, this finding remained stable in the vast majority of subgroups, as SGLT2i was suggested to represent the best interventions among patients with diabetes mellitus, history of cardiovascular disease, eGFR < 45 mL/min/1.73 m^2^, and UACR > 300 mg/g. A similar effect was observed in studies at low risk of bias, designed to randomize exclusively chronic kidney disease patients.

Data regarding the combination of the examined interventions remain limited, as no direct randomized evidence exists. A recent meta-analysis of RCTs has suggested that SGLT2i may exert beneficial cardiovascular and kidney effects irrespective of baseline GLP1a therapy [116]. A similar outcome has been proposed for GLP1a, as its administration is linked to favorable outcomes regardless of baseline SGLT2i intake [117]. On the other hand, observational studies have pointed towards a potential synergistic effect of SGLT2i and GLP1a since their combination has shown superiority over monotherapy with either agent [118,119]. In addition, finerenone has been proposed to exert additive beneficial effects on top of SGLT2i administration [120], representing a potentially cost-effective intervention among diabetic patients [121]. However, clinical trials randomizing patients to combined treatment regimens are needed before safe conclusions can be drawn.

The current KDIGO guidelines recommend SGLT2i treatment for patients with chronic kidney disease, regardless of diabetes mellitus status [12]. Subgroup analyses from large RCTs suggest that the therapeutic benefits of SGLT2i are consistent across various etiologies, including diabetic nephropathy, ischemic, hypertensive, glomerular, and other forms of kidney disease [101,122]. Notably, SGLT2i have shown promise in IgA nephropathy, where their use has been associated with delayed disease progression and a favorable safety profile [123]. Furthermore, in patients with focal segmental glomerulosclerosis, SGLT2i therapy has been linked to a slower decline in eGFR, significant reductions in proteinuria, and decreased cardiovascular mortality risk [124]. However, the efficacy of SGLT2i in autosomal dominant polycystic kidney disease remains unclear due to a lack of RCT evidence. Additionally, concerns regarding potential increases in kidney volume may limit their use in this population [125].

The present study has several strengths. An overview of the existing, partially overlapping network meta-analyses was conducted, highlighting methodological limitations and identifying areas for improvement. An updated network meta-analysis was subsequently conducted, incorporating a large sample size and leveraging the full scope of available randomized evidence. The evidence certainty was rigorously assessed with the CiNeMA tool, enabling a realistic evaluation of the outcomes. The TOPSIS method has also been employed as a novel approach for the determination of the best intervention, simultaneously accounting for multiple outcomes of interest. On the other hand, it is important to note that comparisons among SGLT2i, GLP1a, and finerenone relied solely on indirect evidence, as no head-to-head studies are currently available. As a result, star-shaped networks were generated, precluding the statistical testing of consistency. Drug doses were not considered in this analysis, as the network meta-analysis nodes represented drug categories to enhance statistical power and improve the precision of estimates. No patients were excluded based on the administered dose, as such exclusions could be arbitrary and risk introducing selection bias. Nevertheless, since the analysis focused on phase 3 RCTs, the proportion of patients receiving ineffective doses is expected to be minimal, with a negligible impact on the overall outcomes. Clinical decision-making regarding optimal interventions may be complicated by the heterogeneity of patient characteristics across RCTs. To address this, multiple subgroup analyses were performed, yielding stable results. It is also important to note that the multicriteria decision analysis relied on estimated P-scores, which do not fully capture statistical uncertainty or evidence certainty. Therefore, the treatment selection outcomes should be interpreted within the context of the primary analyses.

## 5. Conclusions

SGLT2i, GLP1a, and ns-MRA are associated with significant reductions in the risk of major cardiovascular events and the progression of kidney disease compared to the placebo, in patients with chronic kidney disease. While indirect evidence suggests that SGLT2i may offer the most favorable balance of efficacy and safety, the certainty of evidence is low, necessitating confirmation through direct comparative trials. Future studies should prioritize head-to-head comparisons of these therapies and investigate the potential for synergistic effects in combination regimens.

## Figures and Tables

**Table 1:** Methodological characteristics of the included network meta-analyses.

Study	Country	Diabetes Mellitus	Nodes	Studies No.	Network Density	Statistical Method	Transitivity Evaluation	Heterogeneity Assessment	Ranking	CoE Evaluation	Funding	Registered Protocol
2024, Kani [36]	USA	Mixed	Ertugliflozin, empagliflozin, dapagliflozin, sotagliflozin, placebo	8	0.4	Frequentist	No	Sensitivity analysis	No	No	Non-profit	Yes
2023, Yang [35]	China	Exclusively	SGLT2i, GLP1a, DPP4i, MRA, atrasentan, pirfenidone, pentoxifylline, placebo	27	0.25	Bayesian	Yes	Subgroup and sensitivity analysis	Yes	Yes	Non-profit	Yes
2022, Cao [34]	China	Exclusively	SGLT2i, GLP1a, DPP4i, placebo	16	0.5	Both	No	Sensitivity analysis	Yes	No	Industry	Yes
2022, Chen [33]	China	Mixed	Dapagliflozin, canagliflozin, sotagliflozin, placebo	3	0.5	Bayesian	No	Subgroup analysis	Yes	No	No	No
2022, Kawai [32]	Japan	Exclusively	SGLT2i, GLP1a, placebo	9	0.67	Frequentist	Yes	Sensitivity analysis	No	No	Non-profit	Yes
2022, Li [31]	China	Exclusively	SGLT2i, GLP1a, DPP4i, placebo	17	0.5	Frequentist	Yes	Subgroup analysis	Yes	No	No	Yes
2022, Lin [30]	China	Exclusively	Canaglifozin (100 and 300 mg), empaglifozin (10 and 25 mg), dapaglifozin (5 and 10 mg), sotagliflozin (200 and 400 mg), luseogliflozin, ipraglifozin, bexaglifozin, ertuglifozin (5 and 15 mg), placebo	14	0.19	Frequentist	No	No	Yes	No	NR	Yes
2020, Malik [29]	USA	Exclusively	Dapagliflozin, canagliflozin, placebo	7	0.5	Frequentist	No	Sensitivity analysis	Yes	No	No	Yes
2023, Morita [28]	Japan	Exclusively	SGLT2i, MRA, MRA + SGLT2i, placebo	17	0.83	Frequentist	No	Sensitivity analysis	No	No	Industry	Yes
2023, Nguyen [44]	Canada	Exclusively	SGLT2i, GLP1a, MRA, placebo	29	0.5	Frequentist	No	Sensitivity analysis	Yes	No	No	Yes
2021, Qiu [43]	China	Exclusively	Dapagliflozin, empagliflozin, canagliflozin, albiglutide, semaglutide, lixisenatide, liraglutide, exenatide, placebo	11	0.18	Bayesian	No	Sensitivity analysis	Yes	No	No	Yes
2022, Qiu [42]	China	Mixed	Dapagliflozin, empagliflozin, canagliflozin, ertugliflozin, sotagliflozin, placebo	10	0.33	Bayesian	No	No	Yes	No	Non-profit	Yes
2022, Tsukamoto [41]	Japan	Exclusively	SGLT2i, MRA, MRA + SGLT2i, placebo	8	1	Frequentist	No	No	No	No	Industry	Yes
2021, Yamada [40]	Japan	Exclusively	SGLT2i, GLP1a, placebo	13	0.67	Frequentist	No	Subgroup and sensitivity analysis	No	No	Non-profit	No
2022, Yang [39]	China	Exclusively	SGLT2i, MRA, placebo	17	0.67	Bayesian	No	No	Yes	Yes	No	Yes
2024, Yang [38]	China	Exclusively	SGLT2i, MRA, placebo	18	0.67	Bayesian	No	No	Yes	No	No	Yes
2022a, Zhang [27]	China	Exclusively	SGLT2i, finerenone, placebo	10	0.67	Frequentist	No	No	No	Yes	Non-profit	Yes
2022b, Zahng [37]	China	Exclusively	SGLT2i, GLP1a, finerenone, placebo	18	0.5	Frequentist	No	Sensitivity analysis	Yes	Yes	Non-profit	Yes
2022, Zhao [26]	China	Exclusively	SGLT2i, finerenone, placebo	14	0.67	Frequentist	No	No	No	No	No	No

CoE, certainty of evidence; SGLT2i, sodium-glucose cotransporter 2 inhibitors; GLP1a, glucagon-like peptide-1 receptor agonist; DPP4i: dipeptidyl peptidase-4 inhibitors; MRA, mineralocorticoid receptor antagonist; NR, not reported.

**Table 2:** Quality assessment of the included network meta-analyses, using the ROBIS and ROB-NMA tools.

	Risk of Bias						
Study	ROBIS			ROB-NMA			Overall
	Study Eligibility Criteria	Identification and Selection of Studies	Data Collection and Study Appraisal	Interventions and Network Geometry	Effect Modification	Synthesis	
2024, Kani [36]	Some concerns	Some concerns	Some concerns	Low	Major concerns	Some concerns	High
2023, Yang [35]	Some concerns	Low	Low	Low	Some concerns	Low	Moderate
2022, Cao [34]	Low	Some concerns	Some concerns	Low	Some concerns	Some concerns	High
2022, Chen [33]	Some concerns	Low	Low	Low	Some concerns	Some concerns	High
2022, Kawai [32]	Low	Low	Some concerns	Low	Some concerns	Low	Moderate
2022, Li [31]	Major concerns	Low	Some concerns	Low	Some concerns	Major concerns	High
2022, Lin [30]	Low	Low	Some concerns	Some concerns	Major concerns	Major concerns	High
2020, Malik [29]	Some concerns	Low	Some concerns	Some concerns	Some concerns	Major concerns	High
2023, Morita [28]	Some concerns	Some concerns	Some concerns	Major concerns	Major concerns	Major concerns	High
2023, Nguyen [44]	Major concerns	Low	Some concerns	Some concerns	Major concerns	Some concerns	High
2021, Qiu [43]	Low	Low	Some concerns	Some concerns	Major concerns	Some concerns	High
2022, Qiu [42]	Some concerns	Low	Some concerns	Low	Major concerns	Major concerns	High
2022, Tsukamoto [41]	Major concerns	Low	Some concerns	Major concerns	Major concerns	Some concerns	High
2021, Yamada [40]	Low	Low	Some concerns	Low	Some concerns	Major concerns	High
2022, Yang [39]	Low	Low	Low	Low	Major concerns	Some concerns	High
2024, Yang [38]	Low	Low	Some concerns	Low	Major concerns	Major concerns	High
2022a, Zhang [27]	Some concerns	Low	Low	Low	Major concerns	Some concerns	High
2022b, Zhang [37]	Some concerns	Low	Low	Low	Major concerns	Some concerns	High
2022, Zhao [26]	Some concerns	Some concerns	Some concerns	Low	Major concerns	Major concerns	High

**Table 3:** Results of the multicriteria decision analysis based on P-scores across five endpoints. Weights for each outcome were assigned both objectively (using the entropy method) and subjectively (using the analytic hierarchy process), considering three distinct clinical scenarios.

Intervention	Equal Weights		Entropy Weights		Scenario 1		Scenario 2		Scenario 3	
	TOPSIS Score	Rank	TOPSIS Score	Rank	TOPSIS Score	Rank	TOPSIS Score	Rank	TOPSIS Score	Rank
SGLT2i	1	1	1	1	1	1	1	1	1	1
GLP1a	0.517	2	0.433	2	0.551	2	0.485	2	0.437	3
ns-MRA	0.429	3	0.354	3	0.503	3	0.427	3	0.321	4
Placebo	0.240	4	0.314	4	0.061	4	0.067	4	0.484	2

In scenario 1, more importance is placed on the cardiovascular composite outcome. In scenario 2, more importance is placed on kidney outcomes (kidney-specific composite outcome and acute kidney injury). In scenario 3, more importance is placed on safety outcomes (serious adverse events and drug discontinuation). Higher TOPSIS scores indicate better interventions. SGLT2i emerged as the best treatment in all scenarios.

## Data Availability

The extracted data are available as Supplementary Materials. All data and analyses are available upon request from the corresponding author.

## Supplementary Materials

The following supporting information can be downloaded at: https://www.mdpi.com/article/10.3390/biom15010039/s1, Figure S1: Search plot diagram; Figure S2: Star-shaped graph of the network meta-analysis; Figure S3: Comparison of age distribution across different interventions (Kruskal-Wallis *p*-value: 0.389); Figure S4: Comparison of female sex percentage across different interventions (Kruskal-Wallis *p*-value: 0.021); Figure S5: Comparison of body mass index distribution across different interventions (Kruskal-Wallis *p*-value: 0.019); Figure S6: Comparison of estimated glomerular filtration rate distribution across different interventions (Kruskal-Wallis *p*-value: 0.165); Figure S7: Comparison of urinary albumin-to-creatinine ratio distribution across different interventions (Kruskal-Wallis *p*-value: 0.520); Figure S8: League table of comparisons in patients with diabetes mellitus; Figure S9: League table of comparisons in patients with cardiovascular disease history; Figure S10: League table of comparisons in patients with chronic kidney disease stage ≥3b; Figure S11: League table of comparisons in patients with chronic kidney disease stage ≥4; Figure S12: League table of comparisons in patients with macroalbuminuria; Figure S13: League table of comparisons in studies at low risk of bias; Table S1: Methodological characteristics of the included randomized controlled trials; Table S2: Definitions of composite endpoints; Table S3: Outcomes of the RoB-2 evaluation; Table S4: Confidence in network meta-analysis for the composite cardiovascular endpoint; Table S5: Confidence in network meta-analysis for the kidney-specific composite endpoint; Table S6: Confidence in network meta-analysis for the risk of any serious adverse event; Table S7: Confidence in network meta-analysis for the risk of drug discontinuation; Table S8: Confidence in network meta-analysis for acute kidney injury risk; Table S9: Determination of weights from the pairwise comparison matrix of analytic hierarchy process.
