# Supplementary material for: Comparative Efficacy and Safety of Cardio-Renoprotective Pharmacological Interventions in Chronic Kidney Disease: An Umbrella Review of Network Meta-Analyses and a Multicriteria Decision Analysis
Source: Biomolecules. 2024 Dec 31;15(1):39. doi: 10.3390/biom15010039 (PMC11764242; doi:10.3390/biom15010039)
Supplement: Supplementary file 1 [file biomolecules-15-00039-s001.zip › biomolecules-3385861-supplementary.pdf]

<u>**Supplementary Section**</u>

Contents

Section S1: PRISMA flowchart 2

Section S2: Methodological characteristics of randomized controlled trials 3

Section S3: Quality assessment of randomized controlled trials 9

Section S4: Transitivity assessment 11

Section S5: Cardiovascular composite outcome 16

Section S6: Kidney-specific composite outcome 17

Section S7: Safety outcomes 18

Section S8: Subgroup analyses 21

Section S9: Analytical hierarchical process 24

Section S1: PRISMA flowchart

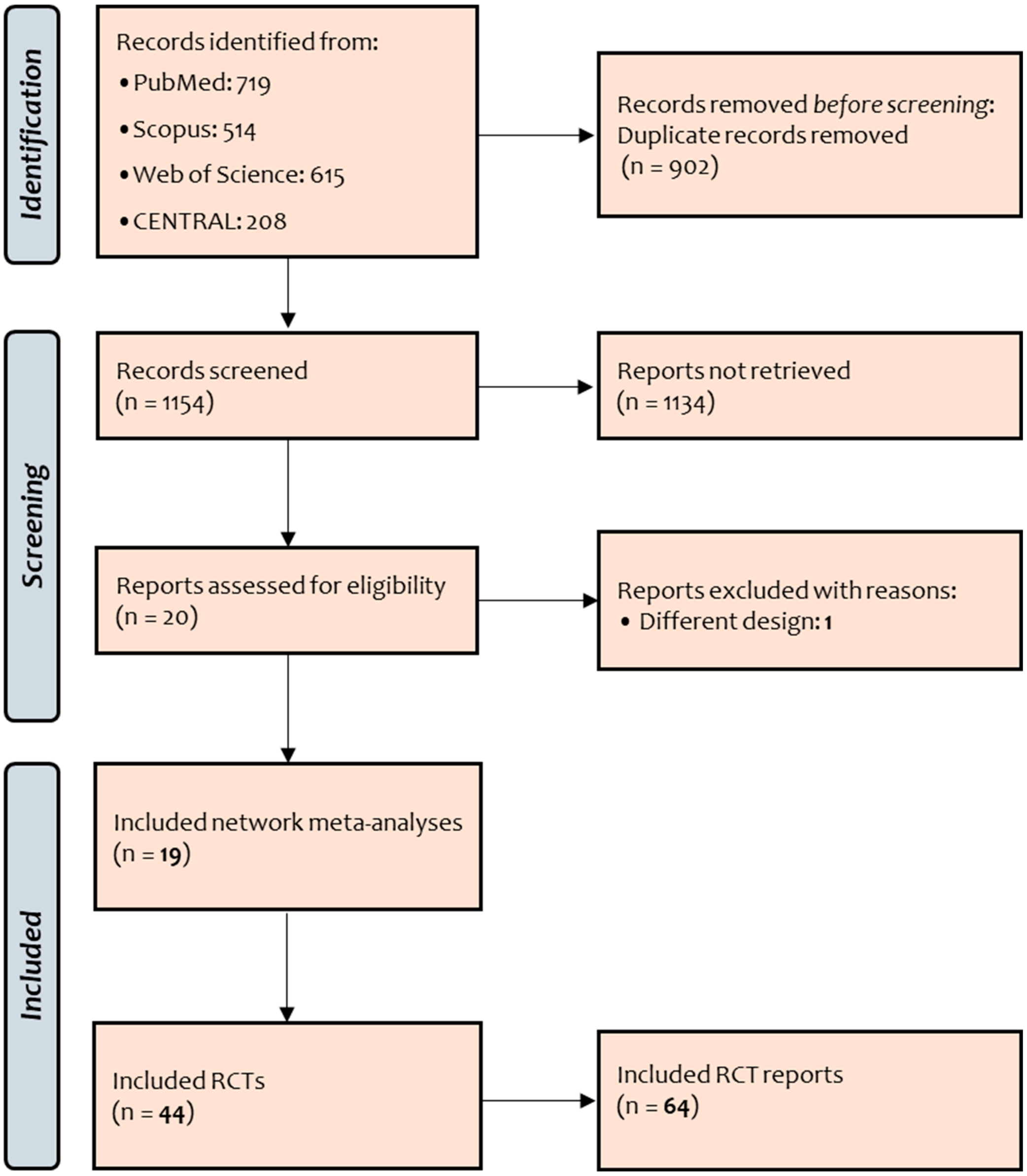

Suppl. Figure S1. Search plot diagram

Section S2: Methodological characteristics of randomized controlled trials

Suppl. Table S1. Methodological characteristics of the included randomized controlled trials.

Year; Author	Trial	CKD	Intervention	Sample size	Age (years)†	Female sex (%)	BMI (kg/m^2)†	Diabetes mellitus (%)	CVD history (%)	eGFR (ml/min/1.73 m^2)†	UACR (mg/g)†
2023; Herrington	EMPA-KIDNEY	Exclusively	Empagliflozin (10 mg)	3304 vs. 3305	63.9 vs. 63.8	33.2 vs. 33.1	29.7 vs. 29.8	46.2 vs. 45.8	26.1 vs. 27.4	37.4 vs. 37.3	219 vs. 226
2020; Cannon; 2021; Cherney; 2021; Dagogo-Jack	VERTIS CV	Subgroup analysis	Ertugliflozin (5/15 mg)	1199 vs. 608	68.3 vs. 68	36.6 vs. 33.9	32.4 vs. 32.6	100	100	49.5 vs. 49	NR
2020; Heerspink; 2022; Waijer; 2021; Wheeler	DAPA-CKD	Exclusively	Dapagliflozin (10 mg)	2152 vs. 2152	61.8 vs. 61.9	32.9 vs. 33.3	29.4 vs. 29.6	67.6 vs. 67.4	37.8 vs. 37.0	43.2 vs. 43.0	965 vs. 934
2024; Perkovic	FLOW	Exclusively	Semaglutide (1 mg)	1767 vs. 1766	66.6 vs. 66.7	29.4 vs. 31.1	31.9 vs. 32.0	100	22.9 vs. 22.8	46.9 vs. 47.1	582 vs. 558
2015; Zinman; 2016; Wanner; 2017; Wanner	EMPA-REG OUTCOME	Subgroup analysis	Empagliflozin (10/25 mg)	1212 vs. 607	67.1 vs. 67.1	32.7 vs. 31.1	31.0 vs. 30.9	100	100	48.4 vs. 48.6	NR
2017; Neal; 2018; Perkovic; 2018; Neuen	CANVAS	Subgroup analysis	Canagliflozin (100/300 mg)	1110 vs. 929	67.6 vs. 67.6	41 vs. 43	31.9 vs. 32.0	100	64.8 vs. 66.7	49.2 vs. 49	21.5 vs. 21.7
2018; Mann; 2020; Mann	LEADER	Subgroup analysis	Liraglutide (1.8 mg)	1116 vs. 1042	67 vs. 67	38 vs. 39	32 vs. 33	100	39 vs. 41	46 vs. 46	47.3 vs. 51.8
2019; Allegretti	NCT02836873	Exclusively	Bexagliflozin (20 mg)	157 vs. 155	69.3 vs. 69.9	41.4 vs. 32.9	30.3 vs. 30.1	100	NR	45.4 vs. 44.8	NR
2021; Anker; 2023; Sharma	EMPEROR-Preserved	Subgroup analysis	Empagliflozin (10 mg)	1615 vs. 1583	74.2 vs. 74.2	48.6 vs. 48.6	30.1 vs. 30.1	52.6 vs. 51.9	100	46.5 vs. 46.1	32 vs. 32
2014; Barnett	EMPA-REG RENAL	Exclusively	Empagliflozin (10/25 mg)	419 vs. 321	63.7 vs. 64.1	40.6 vs. 43.3	30.9 vs. 30.6	100	NR	55.7 vs. 49.9	NR
2021a; Bhatt; 2024; Sridhar	SCORED	Exclusively	Sotagliflozin (200/400 mg)	5292 vs. 5292	69 vs. 69	44.3 vs. 45.5	31.9 vs. 31.7	100	31 vs. 31	44.4 vs. 44.7	74 vs. 75
2021b; Bhatt	SOLOIST-WHF	Subgroup analysis	Sotagliflozin (200/400 mg)	854	NR	NR	NR	100	100	NR	NR
2016; Cherney	NCT01177813; NCT01159600;	Subgroup analysis	Empagliflozin (10/25 mg)	128 vs. 87	59.3 vs. 60.5	32.8 vs. 33.3	29.7 vs. 29.8	100	NR	56.6 vs. 52.5	874 vs. 961

	NCT01210001; EMPA-REG RENAL										
2018; Fioretto	DERIVE	Exclusively	Dapagliflozin (10 mg)	160 vs. 161	66.2 vs. 65.3	43.5 vs. 43.1	31.6 vs. 32.6	100	NR	53.6 vs. 53.3	29 vs. 23.5
2018; Grunberger	VERTIS RENAL	Exclusively	Ertugliflozin (5/15 mg)	313 vs. 154	67.1 vs. 67.5	49.2 vs. 53.2	32.2 vs. 33.2	100	49.9 vs. 49.4	46.8 vs. 46.0	NR
2016; Haneda	JapicCTI-111543	Exclusively	Luseogliflozin (2.5/5 mg)	95 vs. 50	67.9 vs. 68.4	24.2 vs. 22	25.5 vs. 25.8	100	NR	52.0 vs. 52.5	335.7 vs. 231.9
2019; Ito	JapicCTI-152774	Exclusively	Esaxerenone (0.625/1.25/2.5/ 5 mg)	285 vs. 73	65.3 vs. 66	22.1 vs. 22	25.8 vs. 25.9	100	15.8 vs. 19	67.3 vs. 69	109.3 vs. 110
2015; Kashiwagi	LANTERN	Exclusively	Ipragliflozin (50 mg)	118 vs. 46	63.9 vs. 65.7	21.7 vs. 22.0	25.0 vs. 25.8	100	NR	62.7 vs. 60.2	NR
2013; Kohan	MB102029	Exclusively	Dapagliflozin (5/10 mg)	168 vs. 84	67 vs. 67	33.9 vs. 36.9	NR	100	NR	NR	76 vs. 67
2017; Jardine; 2019; Perkovic; 2020; Jardine; 2020; Bakris	CREDENCE	Exclusively	Canagliflozin (100 mg)	2202 vs. 2199	62.9 vs. 63.2	34.6 vs. 33.3	31.4 vs. 31.3	100	50.5 vs. 50.3	56.3 vs. 56.0	923 vs. 931
2019; McMurray; 2021; Jhund	DAPA-HF	Subgroup analysis	Dapagliflozin (10 mg)	962 vs. 964	70.9	27.7	28.4	51.0	100	47	NR
2019; Mosenzon; 2019; Wiviott; 2021; Zelniker	DECLARE-TIMI 58	Subgroup analysis	Dapagliflozin (10 mg)	2944 vs. 2940	64.4	32.4	32.5	100	45.8	80	77.8
2020; Packer; 2021; Zannad	EMPEROR-Reduced	Subgroup analysis	Empagliflozin (10 mg)	981 vs. 987	70.4 vs. 70.1	23.3 vs. 27.4	28.1 vs. 27.9	53.3 vs. 54.4	100	46.5 vs. 47.4	36 vs. 36
2019; Pollock	DELIGHT	Exclusively	Dapagliflozin (10 mg)	145 vs. 148	64.7 vs. 64.7	30 vs. 29	30.2 vs. 30.3	100	54 vs. 44	50.2 vs. 47.7	270 vs. 257.5
2022; Solomon; 2023; McCausland	DELIVER	Subgroup analysis	Dapagliflozin (10 mg)	1516 vs. 1554	74.5 vs. 74.5	48.5 vs. 49.6	29.9 vs. 29.9	49.9 vs. 48.6	100	44.7 vs. 45.0	NR
2022; Voors	EMPULSE	Subgroup analysis	Empagliflozin (10 mg)	161 vs. 145	72.3	38.6	29.6	49.7	100	NR	NR
2022; Wada	NCT03436693	Exclusively	Canagliflozin (100 mg)	154 vs. 154	62.5 vs. 62.4	25.3 vs. 16.2	26.7 vs. 27.1	100	NR	56.3 vs. 55.2	712 vs. 630
2014; Yale	NCT01064414	Exclusively	Canagliflozin (100/300 mg)	179 vs. 90	68.7 vs. 68.2	40.8 vs. 36.7	32.9 vs. 33.1	100	NR	39.1 vs. 40.1	257 vs. 255.1

2020; Bethel	EXSCEL	Subgroup analysis	Exenatide (2 mg)	1557 vs. 1620	66.5	42.9	32.8	100	82.7	49.2	NR
2015; Davies	LIRA-RENAL	Exclusively	Liraglutide (1.8 mg)	140 vs. 139	68.0 vs. 66.3	46.4 vs. 52.6	33.4 vs. 34.5	100	NR	45.4 vs. 45.5	55.5 vs. 69.8
2019; Gerstein; 2023; Botros	REWIND	Subgroup analysis	Dulaglutide (1.5 mg)	944 vs. 988	NR	NR	NR	100	NR	NR	NR
2021; Gerstein	AMPLITUDE-O	Subgroup analysis	Efpeglenatide (4/6 mg)	1371 vs. 666	NR	NR	NR	100	NR	NR	NR
2018; Hernadez	Harmony Outcomes	Subgroup analysis	Albigluide (30/50 mg)	1098 vs. 1124	NR	NR	NR	100	100	NR	NR
2019; Husain	PIONEER 6	Subgroup analysis	Semaglutide (oral 14 mg)	434 vs. 422	NR	NR	NR	100	NR	NR	NR
2023; Tuttle	PIONEER 6; SUSTAIN 6	Subgroup analysis	Semaglutide (oral 14 mg/ sc 1 mg)	779 vs. 781	68.3 vs. 68.8	39 vs. 38	NR	100	NR	47.4 vs. 46.9	48.9 vs. 48.0
2016; Marso	SUSTAIN 6	Subgroup analysis	Semaglutide (sc 1 mg)	469 vs. 470	NR	NR	NR	100	NR	NR	NR
2018; Tuttle	AWARD-7	Exclusively	Dulaglutide (0.75/1.5 mg)	382 vs. 194	64.7 vs. 64.3	45.5 vs. 52	32.6 vs. 33.0	100	NR	38.2 vs. 38.5	223.7 vs. 195.6
2019; Mosenzon	PIONEER 5	Exclusively	Semaglutide (oral 14 mg)	163 vs. 161	71 vs. 70	49 vs. 55	32.2 vs. 32.6	100	NR	47 vs. 48	19.2 vs. 14.1
2015; Pfeffer	ELIXA	Subgroup analysis	Lixisenatide (20 μg)	659 vs. 784	NR	NR	NR	100	100	NR	NR
2015; Bakris	ARTS-DN	Exclusively	Finerenone (1.25-2exc5 mg)	727 vs. 94	64.3 vs. 63.3	21.6 vs. 26.6	31.7 vs. 32.5	100	NR	66.9 vs. 72.2	189.5 vs. 182.9
2020; Bakris; 2020; Filippatos	FIDELIO-DKD	Exclusively	Finerenone (10-20 mg)	2833 vs. 2841	65.4 vs. 65.7	31.1 vs. 28.5	NR	100	NR	44.4 vs. 44.3	833 vs. 867
2021; Pitt	FIGARO-DKD	Exclusively	Finerenone (10-20 mg)	3686 vs. 3666	64.1 vs. 64.1	31.4 vs. 29.7	NR	100	45.5 vs. 45.1	67.6 vs. 68.0	302 vs. 315
2020; Ito	ESAX-DN	Exclusively	Esaxerenone (1.25/2.5 mg)	222 vs. 227	66 vs. 66	21 vs. 26	25.9 vs. 26.2	100	12.2 vs. 10.6	69 vs. 69	113 vs. 110

† Mean values

CKD: chronic kidney disease; BMI: body mass index; CVD: cardiovascular disease; eGFR: estimated glomerular filtration rate; UACR: urinary albumin-to-creatinine ratio; NR: not reported

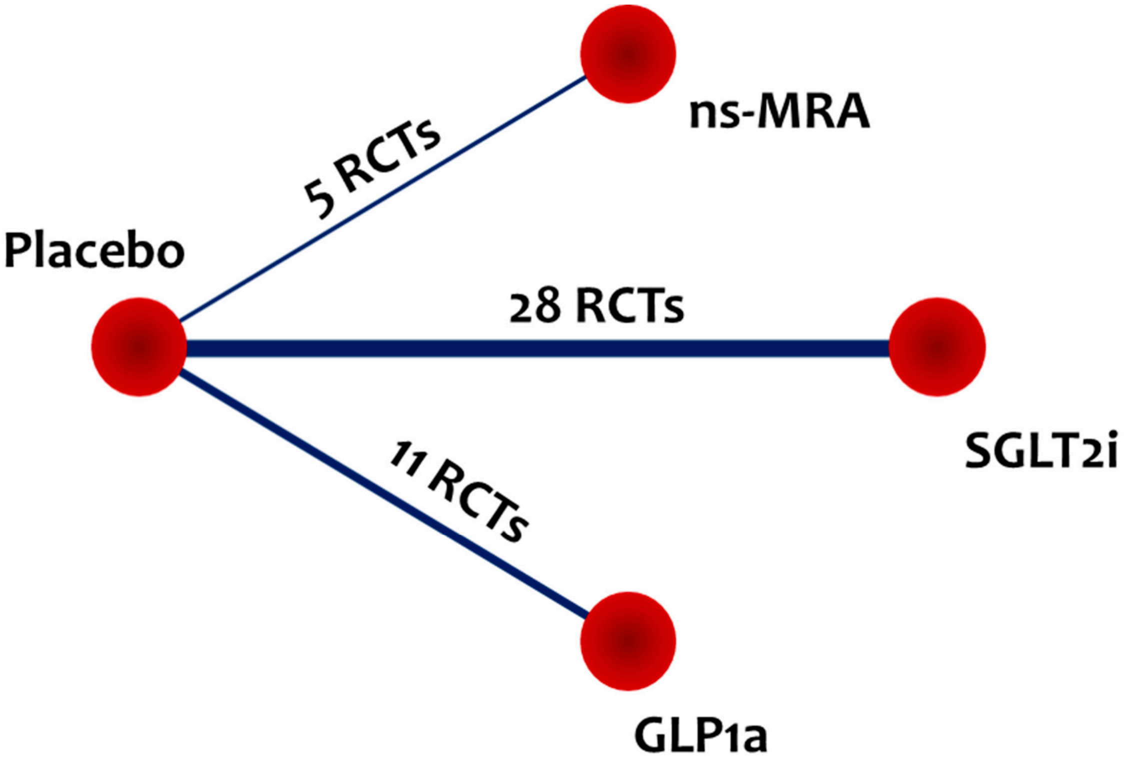

Suppl. Figure S2. Star-shaped graph of the network meta-analysis.

Suppl. Table S2. Definitions of composite endpoints.

Study	Kidney-specific composite outcome	Cardiovascular composite outcome
EMPA-KIDNEY	Composite of kidney failure, a sustained decrease from baseline in the eGFR of at least 40%, or death from renal causes	Composite of hospitalization for heart failure or death from cardiovascular causes
VERTIS CV	Composite of death from renal causes, renal replacement therapy, or doubling of the serum creatinine level	Composite of death from cardiovascular causes, nonfatal myocardial infarction, or nonfatal stroke
DAPA-CKD	Composite of decline in eGFR of ≥50%, end-stage kidney disease, or death from renal causes	Composite of death from cardiovascular causes or hospitalization for heart failure
FLOW	Composite of kidney failure, a sustained (for ≥28 days) 50% or greater reduction in eGFR from baseline, or death from kidney-related causes	Composite of nonfatal myocardial infarction, nonfatal stroke, or death from cardiovascular causes
EMPA-REG OUTCOME	Composite of a doubling of the serum creatinine level, initiation of renal-replacement therapy, or death from renal disease	Composite of death from cardiovascular causes, nonfatal myocardial infarction (excluding silent myocardial infarction), or nonfatal stroke
CANVAS	Composite of doubling of serum creatinine, end-stage kidney disease, or death from renal causes	Composite of death from cardiovascular causes, nonfatal myocardial infarction, or nonfatal stroke
LEADER	Composite of new-onset persistent macroalbuminuria, persistent doubling of the serum creatinine level and an estimated GFR of 45 ml or less per minute per 1.73 m^2, need for continuous renal-replacement therapy with no reversible cause of the renal disease, or death from renal disease	Composite cardiovascular death, nonfatal (including silent) myocardial infarction, or nonfatal stroke
2019; Allegretti	-	NR
EMPEROR-Preserved	Composite of chronic dialysis or kidney transplant or sustained reduction of ≥40% in eGFR or sustained eGFR<15ml/min/1.73m^2 (for patients with baseline eGFR ≥30) or sustained eGFR <10 (for patients baseline eGFR<30)	Composite of death from cardiovascular causes or hospitalization for heart failure
EMPA-REG RENAL	-	-
SCORED	Composite of sustained decrease of ≥50% in the eGFR from baseline for ≥30 days, long-term dialysis, renal transplantation, or sustained eGFR of <15 ml/min/1.73 m^2 for ≥30 days	Composite of death from cardiovascular causes, hospitalizations for heart failure, and urgent visits for heart failure
SOLOIST-WHF	-	Composite of death from cardiovascular causes, and hospitalization or urgent visit for heart failure
CREDENCE	Composite of end-stage kidney disease, doubling of the serum creatinine level, or renal death	Composite of cardiovascular death or hospitalization for heart failure
DAPA-HF		Composite of worsening heart failure or death from cardiovascular causes
DECLARE-TIMI 58	Composite of sustained decrease in eGFR by at least 40% to less than 60 mL/min per 1.73 m², end-stage kidney disease, or renal death.	Composite of cardiovascular death or hospitalization for heart failure
EMPEROR-Reduced	Composite of chronic dialysis or kidney transplant or sustained reduction of ≥40% in eGFR or sustained eGFR<15ml/min/1.73m^2 (for patients with baseline eGFR ≥30) or sustained eGFR <10 (for patients with baseline eGFR<30)	Composite of cardiovascular death or hospitalization for heart failure
DELIGHT	-	-

DELIVER	Composite of a sustained 50% or greater decline in eGFR relative to baseline, development of end-stage kidney disease, or death due to kidney causes	Composite of worsening heart failure or death from cardiovascular causes
2016; Cherney	-	-
EMPULSE	-	Composite of cardiovascular death or heart failure event
NCT03436693	Composite of end-stage kidney disease, doubling of serum creatinine or renal death	Composite of cardiovascular death or hospitalization for heart failure
EXSCEL	Composite of 40%decline in eGFR, renal replacement or renal death	Composite of cardiovascular death, nonfatal myocardial infarction, or nonfatal stroke
REWIND	Composite ≥40% sustained eGFR decline, end-stage kidney disease, or renal-related death	-
AMPLITUDE-O	-	Composite of nonfatal myocardial infarction, nonfatal stroke, or death from cardiovascular or undetermined causes
Harmony Outcomes	-	Composite of death from cardiovascular causes, myocardial infarction, or stroke
PIONEER 6	-	Composite of death from cardiovascular causes (including undetermined causes of death), nonfatal myocardial infarction, or nonfatal stroke
2023; Tuttle	≥50% sustained eGFR decline	-
SUSTAIN 6	-	Composite of death from cardiovascular causes, myocardial infarction, or stroke
ELIXA	-	Composite of death from cardiovascular causes, nonfatal myocardial infarction, nonfatal stroke, or hospitalization for unstable angina
FIDELIO-DKD	Composite of kidney failure, a sustained decrease of ≥40% in the eGFR from baseline, or death from renal causes	Composite of death from cardiovascular causes, nonfatal myocardial infarction, nonfatal stroke, or hospitalization for heart failure
FIGARO-DKD	Composite of kidney failure, a sustained decrease of ≥40% in the eGFR from baseline for at least 4 weeks, or death from renal causes	Composite of death from cardiovascular causes, nonfatal myocardial infarction, nonfatal stroke, or hospitalization for heart failure

eGFR: estimated glomerular filtration rate; NR: not reported

Section S3: Quality assessment of randomized controlled trials

Suppl. Table S3. Outcomes of the RoB-2 evaluation.

Trial	Risk of bias					
	Randomization process	Deviations from intended interventions	Missing outcome data	Measurement of the outcome	Selection of the reported result	Overall
EMPA-KIDNEY	Low	Low	Low	Low	Low	Low
VERTIS CV	Some concerns	Low	Low	Low	Low	Some concerns
DAPA-CKD	Low	Low	Low	Low	Low	Low
FLOW	Low	Low	Low	Low	Low	Low
EMPA-REG OUTCOME	Some concerns	Low	Low	Low	Low	Some concerns
CANVAS	Some concerns	Low	Low	Low	Low	Some concerns
LEADER	Some concerns	Low	Low	Low	Low	Some concerns
NCT02836873	Low	Low	Low	Low	Low	Low
EMPEROR-Preserved	Some concerns	Low	Low	Low	Low	Some concerns
EMPA-REG RENAL	Low	Low	Low	Low	Low	Low
SCORED	Low	Low	Low	Low	Low	Low
SOLOIST-WHF	Some concerns	Low	Low	Low	Some concerns	Some concerns
NCT01177813	Some concerns	Low	Low	Low	Low	Some concerns
NCT01159600	Some concerns	Low	Low	Low	Low	Some concerns
NCT01210001	Some concerns	Low	Low	Low	Low	Some concerns
DERIVE	Some concerns	Low	Low	Low	Low	Some concerns
VERTIS RENAL	Low	Low	Low	Low	Low	Low
JapicCTI-111543	Low	Low	Low	Low	Low	Low
JapicCTI-152774	Some concerns	Low	Low	Low	Low	Some concerns
LANTERN	Low	Low	Low	Low	Low	Low

MB102029	Some concerns	Low	Low	Low	Low	Some concerns
CREDENCE	Low	Low	Low	Low	Low	Low
DAPA-HF	Some concerns	Low	Low	Low	Low	Some concerns
DECLARE-TIMI 58	Some concerns	Low	Low	Low	Low	Some concerns
EMPEROR-Reduced	Some concerns	Low	Low	Low	Low	Some concerns
DELIGHT	Low	Low	Low	Low	Low	Low
DELIVER	Some concerns	Low	Low	Low	Low	Some concerns
EMPULSE	Some concerns	Low	Low	Low	Low	Some concerns
NCT03436693	Low	Low	Low	Low	Low	Low
NCT01064414	Low	Low	Low	Low	Low	Low
EXSCEL	Some concerns	Low	Low	Low	Low	Some concerns
LIRA-RENAL	Low	Low	Low	Low	Low	Low
REWIND	Some concerns	Low	Low	Low	Low	Some concerns
AMPLITUDE-O	Some concerns	Low	Low	Low	Low	Some concerns
Harmony Outcomes	Some concerns	Low	Low	Low	Low	Some concerns
PIONEER 6	Some concerns	Low	Low	Low	Low	Some concerns
SUSTAIN 6	Some concerns	Low	Low	Low	Low	Some concerns
AWARD-7	Low	Some concerns	Low	Low	Low	Some concerns
PIONEER 5	Low	Low	Low	Low	Low	Low
ELIXA	Some concerns	Low	Low	Low	Low	Some concerns
ARTS-DN	Some concerns	Low	Low	Low	Low	Some concerns
FIDELIO-DKD	Low	Low	Low	Low	Low	Low
FIGARO-DKD	Low	Low	Low	Low	Low	Low
ESAX-DN	Some concerns	Low	Low	Low	Low	Some concerns

Section S4: Transitivity assessment

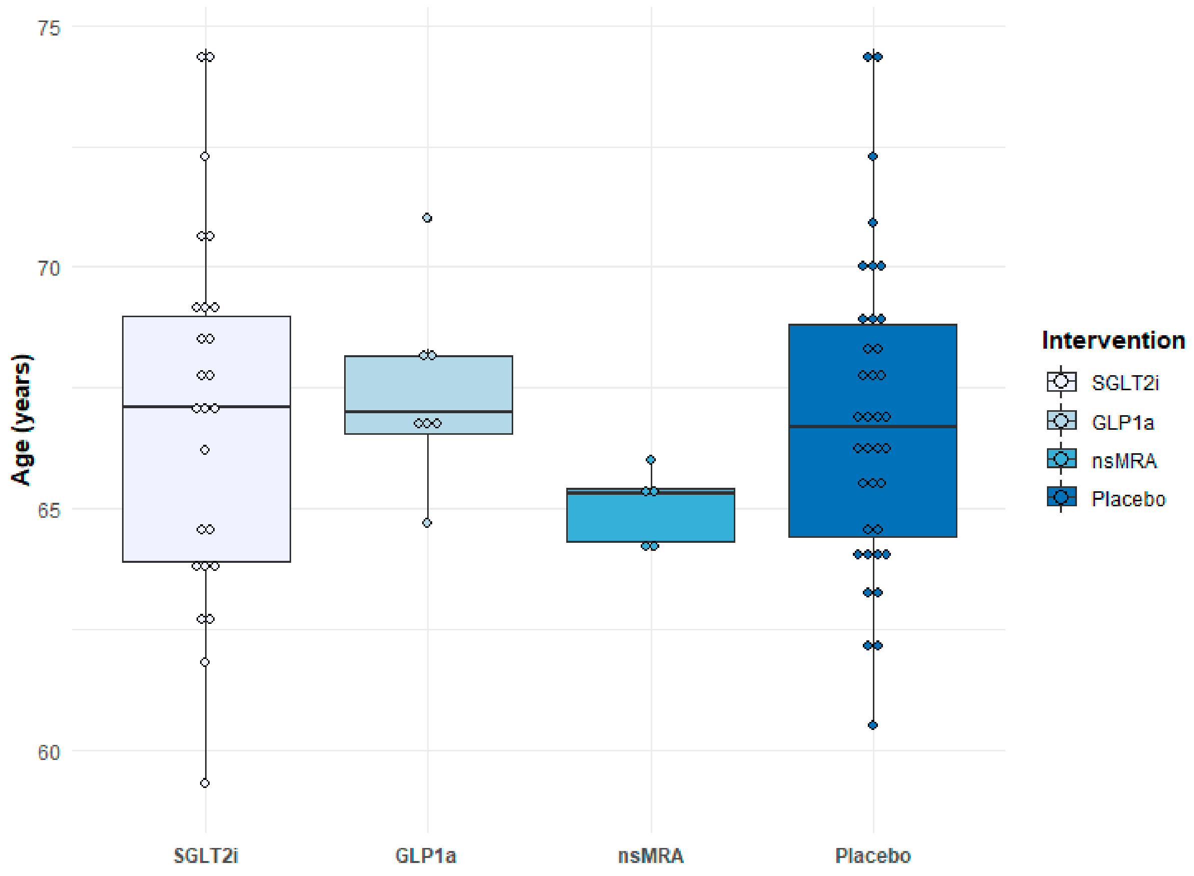

Suppl. Figure S3. Comparison of age distribution across different interventions (Kruskal-Wallis *p-value*: 0.389).

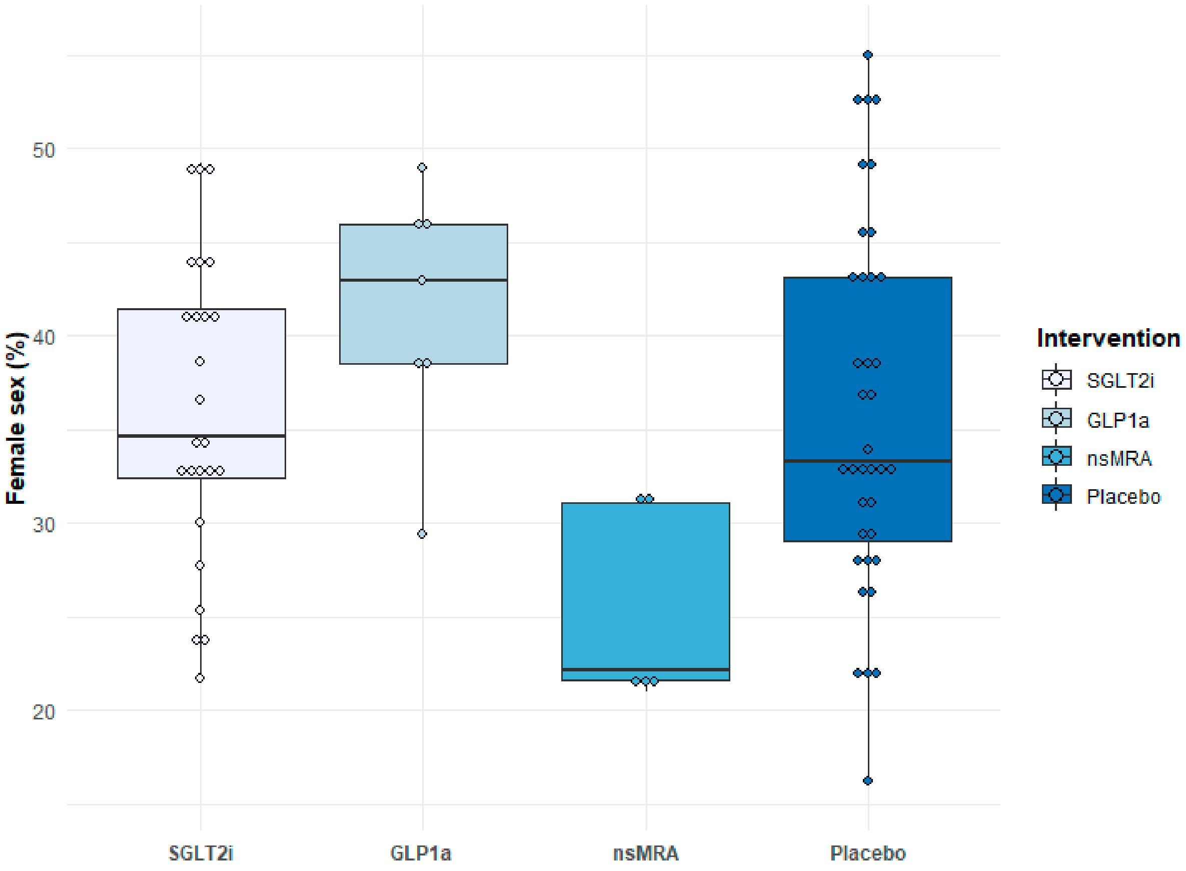

Suppl. Figure S4. Comparison of female sex percentage across different interventions (Kruskal-Wallis *p-value*: 0.021).

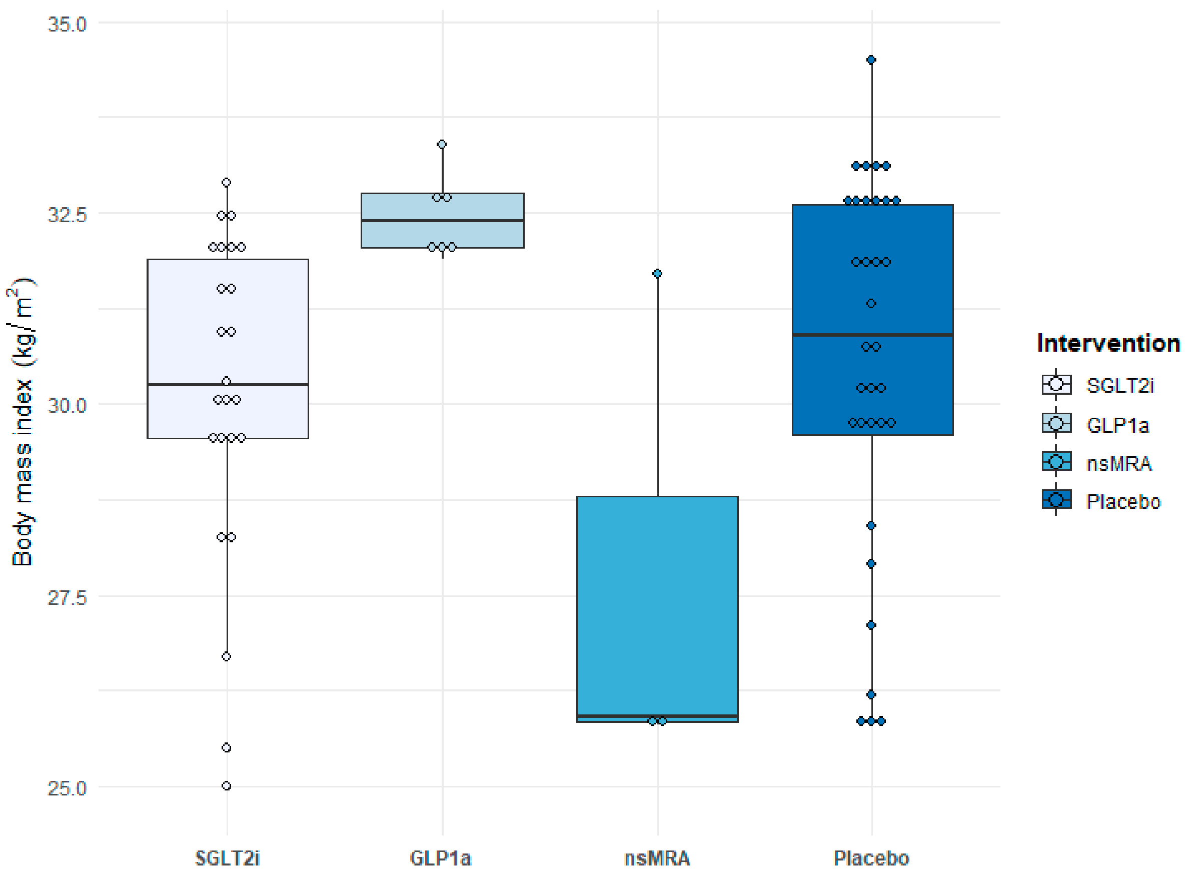

Suppl. Figure S5. Comparison of body mass index distribution across different interventions (Kruskal-Wallis *p-value*: 0.019).

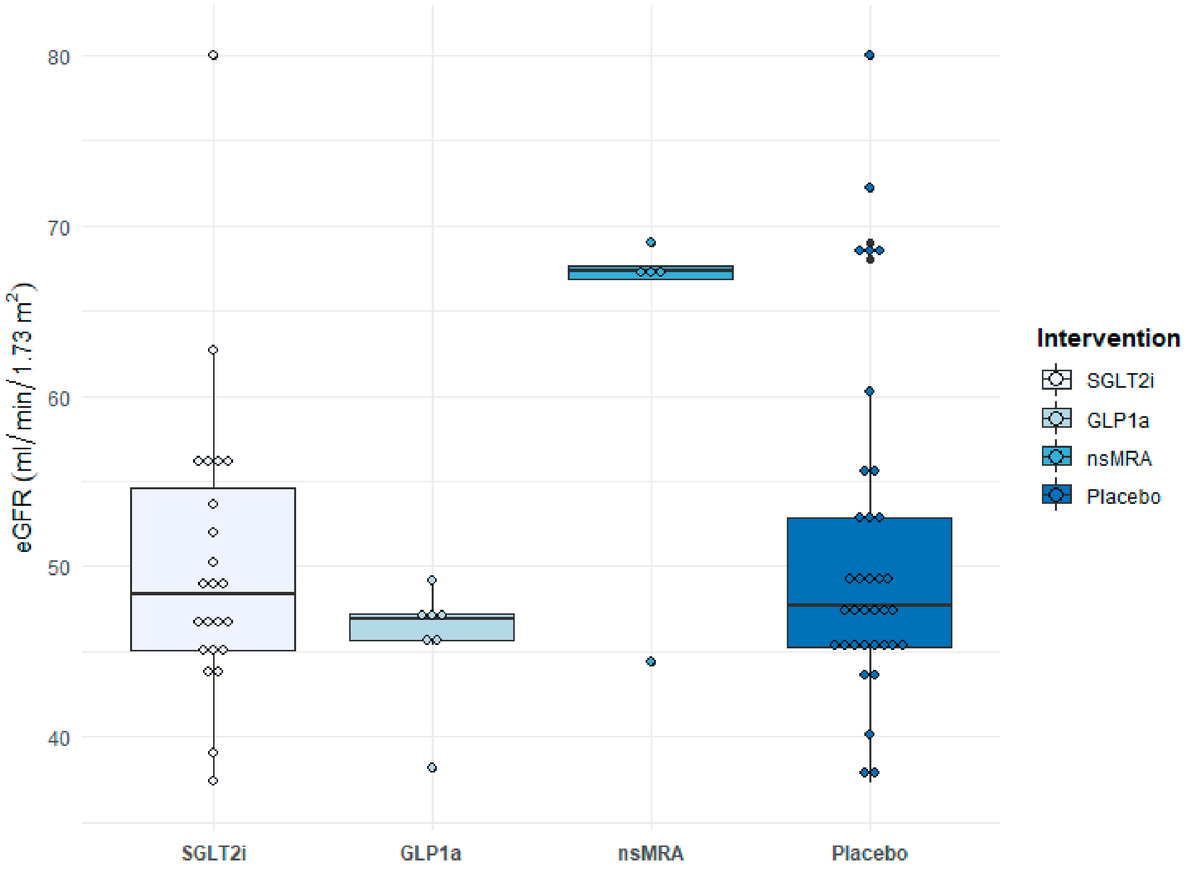

Suppl. Figure S6. Comparison of estimated glomerular filtration rate distribution across different interventions (Kruskal-Wallis *p-value*: 0.165).

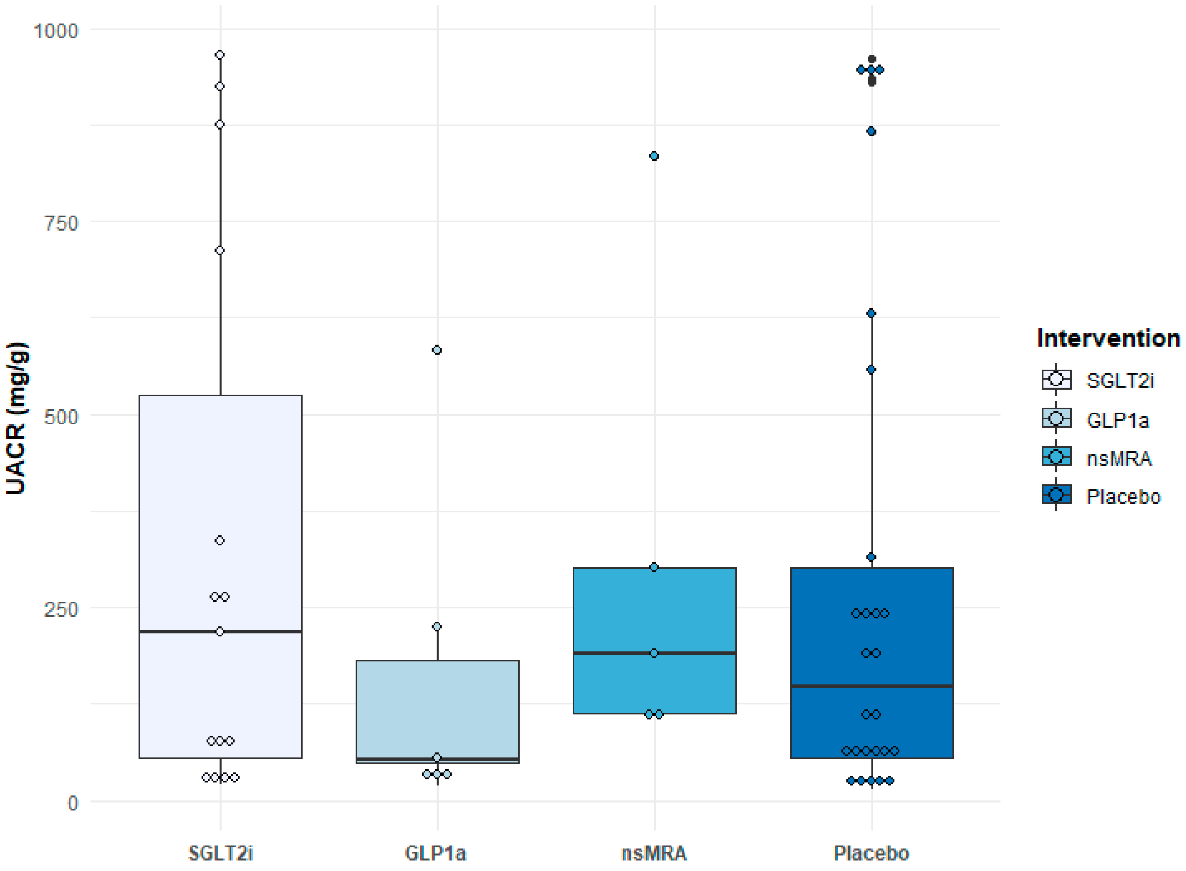

Suppl. Figure S7. Comparison of urinary albumin-to-creatinine ratio distribution across different interventions (Kruskal-Wallis *p-value*: 0.520).

Section S5: Cardiovascular composite outcome

Suppl. Table S4. Confidence in network meta-analysis for the composite cardiovascular endpoint.

Comparison	Within-study bias	Reporting bias	Indirectness	Imprecision	Heterogeneity	Incoherence	Confidence
			**Direct evidence**				
SGLT2i vs. Placebo	Some concerns	Low risk	No concerns	No concerns	Some concerns	No concerns	Low
GLP1a vs. Placebo	Some concerns	Low risk	No concerns	No concerns	No concerns	No concerns	Moderate
nsMRA vs. Placebo	No concerns	Low risk	No concerns	No concerns	Some concerns	No concerns	Moderate
			**Indirect evidence**				
SGLT2i vs. GLP1a	Some concerns	Low risk	No concerns	Some concerns	Some concerns	Some concerns	Low
GLP1a vs. nsMRA	No concerns	Low risk	No concerns	Major concerns	No concerns	Some concerns	Very low
SGLT2i vs. nsMRA	No concerns	Low risk	No concerns	Some concerns	Some concerns	Some concerns	Low

Section S6: Kidney-specific composite outcome

Suppl. Table S5. Confidence in network meta-analysis for the kidney-specific composite endpoint.

Comparison	Within-study bias	Reporting bias	Indirectness	Imprecision	Heterogeneity	Incoherence	Confidence
			**Direct evidence**				
SGLT2i vs. Placebo	Some concerns	Low risk	No concerns	No concerns	No concerns	Some concerns	Low
GLP1a vs. Placebo	No concerns	Low risk	No concerns	No concerns	No concerns	No concerns	High
nsMRA vs. Placebo	No concerns	Low risk	No concerns	No concerns	No concerns	Major concerns	Low
			**Indirect evidence**				
SGLT2i vs. GLP1a	Some concerns	Low risk	No concerns	Some concerns	Some concerns	Some concerns	Low
GLP1a vs. nsMRA	No concerns	Low risk	No concerns	Major concerns	No concerns	Some concerns	Very low
SGLT2i vs. nsMRA	No concerns	Low risk	No concerns	No concerns	Some concerns	Some concerns	Low

Section S7: Safety outcomes

Suppl. Table S6. Confidence in network meta-analysis for the risk of any serious adverse event.

Comparison	Within-study bias	Reporting bias	Indirectness	Imprecision	Heterogeneity	Incoherence	Confidence
			**Direct evidence**				
SGLT2i vs. Placebo	No concerns	Low risk	No concerns	No concerns	Major concerns	No concerns	Low
GLP1a vs. Placebo	No concerns	Low risk	No concerns	Major concerns	No concerns	No concerns	Low
nsMRA vs. Placebo	No concerns	Low risk	No concerns	Major concerns	No concerns	No concerns	Low
			**Indirect evidence**				
SGLT2i vs. GLP1a	No concerns	Low risk	No concerns	Some concerns	Some concerns	Some concerns	Low
GLP1a vs. nsMRA	No concerns	Low risk	No concerns	Major concerns	No concerns	Some concerns	Very low
SGLT2i vs. nsMRA	No concerns	Low risk	No concerns	Major concerns	No concerns	Some concerns	Very low

Suppl. Table S7. Confidence in network meta-analysis for the risk of drug discontinuation.

Comparison	Within-study bias	Reporting bias	Indirectness	Imprecision	Heterogeneity	Incoherence	Confidence
			**Direct evidence**				
SGLT2i vs. Placebo	Some concerns	Low risk	No concerns	Major concerns	No concerns	No concerns	Very low
GLP1a vs. Placebo	No concerns	Low risk	No concerns	Some concerns	Some concerns	No concerns	Low
nsMRA vs. Placebo	No concerns	Low risk	No concerns	Major concerns	No concerns	No concerns	Low
			**Indirect evidence**				
SGLT2i vs. GLP1a	Some concerns	Low risk	No concerns	Some concerns	Some concerns	Some concerns	Low
GLP1a vs. nsMRA	No concerns	Low risk	No concerns	Major concerns	No concerns	Some concerns	Very low
SGLT2i vs. nsMRA	No concerns	Low risk	No concerns	Some concerns	Some concerns	Some concerns	Low

Suppl. Table S8. Confidence in network meta-analysis for acute kidney injury risk.

Comparison	Within-study bias	Reporting bias	Indirectness	Imprecision	Heterogeneity	Incoherence	Confidence
**Direct evidence**							
SGLT2i vs. Placebo	No concerns	Low risk	No concerns	No concerns	No concerns	No concerns	High
GLP1a vs. Placebo	No concerns	Low risk	No concerns	Major concerns	No concerns	No concerns	Low
nsMRA vs. Placebo	No concerns	Low risk	No concerns	Major concerns	No concerns	No concerns	Low
**Indirect evidence**							
SGLT2i vs. GLP1a	No concerns	Low risk	No concerns	Some concerns	No concerns	Some concerns	Low
GLP1a vs. nsMRA	No concerns	Low risk	No concerns	Major concerns	No concerns	Some concerns	Very low
SGLT2i vs. nsMRA	No concerns	Low risk	No concerns	Some concerns	No concerns	Some concerns	Low

Section S8: Subgroup analyses

**SGLT2i**	0.762 (0.655-0.886) *	0.745 (0.654-0.850) *	0.627 (0.571-0.688) *
0.904 (0.779-1.048)	**GLP1a**	0.979 (0.842-1.138)	0.823 (0.730-0.927) *
0.888 (0.733-1.076)	0.983 (0.804-1.201)	**nsMRA**	0.841 (0.767-0.922) *
0.768 (0.698-0.846) *	0.850 (0.760-0.951) *	0.865 (0.733-1.022)	**Placebo**

Cardiovascular composite outcome

Kidney-specific composite outcome

Suppl. Figure S8. League table of comparisons in patients with diabetes mellitus.

**SGLT2i**	NA	NA	0.818 (0.624-1.072)
0.817 (0.642-1.039)	**GLP1a**	NA	NA
NA	NA	**nsMRA**	NA
0.793 (0.717-0.877) *	0.971 (0.780-1.208)	NA	**Placebo**

Cardiovascular composite outcome

Kidney-specific composite outcome

Suppl. Figure S9. League table of comparisons in patients with cardiovascular disease history. *NA: not applicable*

**SGLT2i**	NA	0.874 (0.662-1.550)	0.717 (0.631-0.815) *
0.745 (0.560-0.989) *	**GLP1a**	NA	NA
0.872 (0.731-1.040)	1.171 (0.859-1.595)	**nsMRA**	0.820 (0.712-0.945) *
0.801 (0.732-0.878) *	1.076 (0.822-1.409)	0.919 (0.790-1.070)	**Placebo**

Cardiovascular composite outcome

Kidney-specific composite outcome

Suppl. Figure S10. League table of comparisons in patients with chronic kidney disease stage ≥3b. *NA: not applicable*

**SGLT2i**	NA	NA	0.701 (0.607-0.810) *
1.063 (0.384-2.944)	**GLP1a**	NA	NA
1.802 (0.828-3.924)	1.696 (0.490-5.872)	**nsMRA**	NA
0.776 (0.619-0.972) *	0.730 (0.270-1.92)	0.430 (0.204-0.906) *	**Placebo**

Cardiovascular composite outcome

Kidney-specific composite outcome

Suppl. Figure S11. League table of comparisons in patients with chronic kidney disease stage ≥4. *NA: not applicable*

**SGLT2i**	NA	0.784 (0.658-0.933) *	0.629 (0.562-0.918) *
0.730 (0.550-0.970) *	**GLP1a**	NA	NA
0.761 (0.643-0.899) *	1.041 (0.786-1.381)	**nsMRA**	0.802 (0.701-0.905) *
0.665 (0.589-0.750) *	0.910 (0.704-1.118)	0.874 (0.778-0.982) *	**Placebo**

Cardiovascular composite outcome

Kidney-specific composite outcome

Suppl. Figure S12. League table of comparisons in patients with macroalbuminuria. *NA: not applicable*

**SGLT2i**	0.830 (0.677-1.017)	0.779 (0.681-0.892) *	0.655 (0.596-0.721) *
0.900 (0.730-1.109)	**GLP1a**	0.940 (0.766-1.152)	0.790 (0.660-0.946) *
0.853 (0.743-0.979) *	0.948 (0.772-1.164)	**nsMRA**	0.841 (0.764-0.925) *
0.738 (0.667-0.816) *	0.820 (0.683-0.984) *	0.865 (0.788-0.951) *	**Placebo**

Cardiovascular composite outcome

Kidney-specific composite outcome

Suppl. Figure S13. League table of comparisons in studies at low risk of bias.

Section S9: Analytical hierarchical process

Suppl. Table S9. Determination of weights from the pairwise comparison matrix of analytic hierarchy process.

Criteria	Cardiovascular composite outcome	Kidney-specific composite outcome	Acute kidney injury	Serious adverse event	Drug discontinuation	Weight
Scenario 1						
Cardiovascular composite outcome	1	3	5	9	7	0.511
Kidney-specific composite outcome	1/3	1	3	7	5	0.267
Acute kidney injury	1/5	1/3	1	4	2	0.118
Serious adverse event	1/9	1/7	1/4	1	1/2	0.039
Drug discontinuation	1/7	1/5	1/2	2	1	0.065
**Consistency ratio**						**2.8%**
Scenario 2						
Cardiovascular composite outcome	1	1/5	1/5	5	3	0.131
Kidney-specific composite outcome	5	1	2	8	6	0.348
Acute kidney injury	5	1/2	1	7	5	0.304
Serious adverse event	1/5	1/8	1/7	1	1/2	0.087
Drug discontinuation	1/3	1/6	1/5	2	1	0.043
**Consistency ratio**						**7.0%**
Scenario 3						
Cardiovascular composite outcome	1	1/2	1/5	1/9	1/7	0.037
Kidney-specific composite outcome	2	1	1/3	1/9	1/7	0.053
Acute kidney injury	5	3	1	1/3	1/4	0.138
Serious adverse event	7	7	3	1	1/2	0.303
Drug discontinuation	9	9	4	2	1	0.468
**Consistency ratio**						**2.7%**

In scenario 1, more importance is placed on the cardiovascular composite outcome.
In scenario 2, more importance is placed on kidney outcomes (kidney-specific composite outcome and acute kidney injury).
In scenario 3, more importance is placed on safety outcomes (serious adverse events and drug discontinuation).
